# Safety, tolerability, and clinical and neural effects of single-dose psilocybin in obsessive–compulsive disorder: protocol for a randomized, double-blind, placebo-controlled, non-crossover trial

**DOI:** 10.3389/fpsyt.2023.1178529

**Published:** 2023-04-25

**Authors:** Terence H. W. Ching, Rachael Grazioplene, Calvin Bohner, Stephen A. Kichuk, Giuliana DePalmer, Elizabeth D’Amico, Jeffrey Eilbott, Anastasia Jankovsky, Michelle Burke, Jamila Hokanson, Brad Martins, Chelsea Witherow, Prerana Patel, Lucia Amoroso, Henry Schaer, Christopher Pittenger, Benjamin Kelmendi

**Affiliations:** ^1^Department of Psychiatry, Yale University School of Medicine, New Haven, CT, United States; ^2^Department of Psychology, Yale University, New Haven, CT, United States; ^3^Center for Brain and Mind Health, Yale University School of Medicine, New Haven, CT, United States; ^4^Child Study Center, Yale University School of Medicine, New Haven, CT, United States

**Keywords:** psilocybin, psychedelic, psychological support, obsessive–compulsive disorder, mental health, neuroimaging, adult psychiatry

## Abstract

**Background:**

Psilocybin may help treat obsessive–compulsive disorder (OCD). To date, only one open-label study of psilocybin for OCD exists, necessitating further investigation with a randomized controlled design. The neural correlates of psilocybin’s effects on OCD have also not been studied.

**Objectives:**

This first-of-its-kind trial aims to evaluate the feasibility, safety, and tolerability of psilocybin in the treatment of OCD, provide preliminary evidence on the effects of psilocybin on OCD symptoms, and elucidate neural mechanisms that may mediate psilocybin’s effects on OCD.

**Design:**

We use a randomized (1:1), double-blind, placebo-controlled, non-crossover design to examine the clinical and neural effects of either a single dose of oral psilocybin (0.25 mg/kg) or active placebo-control agent (250 mg of niacin) on OCD symptoms.

**Methods and analysis:**

We are enrolling 30 adult participants at a single site in Connecticut, USA who have failed at least one trial of standard care treatment (medication/psychotherapy) for OCD. All participants will also receive unstructured, non-directive psychological support during visits. Aside from safety, primary outcomes include OCD symptoms over the past 24 h, assessed by the Acute Yale-Brown Obsessive–Compulsive Scale and Visual Analog Scale ratings. These are collected by blinded, independent raters at baseline and the primary endpoint of 48 h post-dosing. Total follow-up is 12 weeks post-dosing. Resting state neuroimaging data will be collected at baseline and primary endpoint. Participants randomized to placebo will be offered the chance to return for an open-label dose of 0.25 mg/kg.

**Ethics statement:**

All participants will be required to provide written informed consent. The trial (protocol v. 5.2) was approved by the institutional review board (HIC #2000020355) and registered with ClinicalTrials.gov (NCT03356483).

**Discussion:**

This study may represent an advance in our ability to treat refractory OCD, and pave the way for future studies of neurobiological mechanisms of OCD that may respond to psilocybin.

## 1. Introduction

### 1.1. Background and rationale

Obsessive–compulsive disorder (OCD) has a lifetime (to date) prevalence and morbid risk of 2.3 and 2.7%, respectively (1). OCD is characterized by obsessions (recurrent, intrusive thoughts, images, or impulses that induce significant anxiety or distress) and compulsions (repetitive and/or ritualized physical or mental actions undertaken in an attempt to reduce that anxiety or distress). As OCD progresses, it is often disabling and chronic (2–4), partly due to long latencies to treatment (5).

If treated, selective serotonin reuptake inhibitors (SSRIs) are the first-line pharmacological option. Although SSRIs have been shown to be efficacious across randomized placebo-controlled trials (6), most patients do not show significant response until at least 2 weeks of continuous treatment (7), and up to 60% of patients do not respond (8–10). Cognitive-behavior therapy (CBT) with exposure and response prevention (ERP) is the first-line psychological treatment (11–14). However, access to appropriately trained therapists remains limited (15–19). Further, many OCD patients classified as responders in SSRI and CBT/ERP trials remain symptomatic and lead restricted lives (20). Relapse after SSRI discontinuation or completion of CBT is also common (21, 22). While neurosurgery is an option for severe, treatment-refractory OCD (23), fundamentally novel, faster-acting, and less invasive alternatives are urgently needed.

Psilocybin is a tryptamine alkaloid prodrug found naturally in mushrooms of the genus *Psilocybe*. When ingested, psilocybin is metabolized into psilocin, which is an agonist or partial agonist at several serotonin receptors, with complex downstream effects (24, 25). Acute psychological effects of psilocin action include altered visuospatial, motion, and time perception, changes in information processing and thought content, depersonalization and derealization, dizziness, impaired concentration, profound positive and negative emotions and mood lability, and paresthesia (26). Common acute physiological effects are related to sympathetic nervous system activation (e.g., changes in body temperature, increases in blood pressure or heart rate) (27, 28). These effects, both psychological and physiological, are transient, typically lasting no longer than 6 h after psilocybin administration (26, 28). The majority of individuals receiving psilocybin in controlled settings maintain insight into the nature and source of their experiences. No convincing evidence exists for symptoms of hallucinogen persisting perception disorder (HPPD) when psilocybin is administered in controlled research settings. Additionally, instances of serious adverse events (SAEs) have generally been low in these settings (29, 30).

Psilocybin dosing, paired with unstructured psychological support, has been examined in clinical trials for treatment-resistant depression, cancer-related anxiety and depression, alcohol dependence, and tobacco addiction (31). Early trials (*n* = 4) used an open-label design, while more recent trials have used a randomized controlled design (*n* = 7) (31). To date, aside from a few case reports (32–34), there has only been one small study of psilocybin in OCD (35). In that study, nine participants were each assigned to receive a dose of oral psilocybin paired with unstructured psychological support per week for 4 weeks: low (100 μg/kg), medium (200 μg/kg), then high (300 μg/kg) dose in that order, with a very low dose (25 μg/kg) randomly inserted, in a double-blind fashion, at some point after the first dose. That study was designed primarily to assess safety and tolerability, but clinical data were also collected. There was a significant main effect of time on OCD symptoms, with 23 to 100% reduction in Yale-Brown Obsessive Compulsive Scale (Y-BOCS) (36, 37) scores 24 h after dosing, well beyond both the expected pharmacological life of psilocybin. Other than transient hypertension in one participant, no SAEs were observed. One participant maintained full symptom remission at 6-month follow-up. The authors speculated on the mechanisms underlying the effects of psilocybin on acute and longer-term OCD symptom improvement, including transcendental experiences that catalyze significant acute feelings of wellness, rapid 5-HT receptor down-regulation, and sustained, adaptive changes in metacognitive processes (e.g., attention, working memory). These preliminary findings justify investigating the feasibility, tolerability, safety, and potential therapeutic effects of psilocybin on OCD more rigorously with a randomized, double-blind, placebo-controlled design. We present such a trial here.

An additional aim of the present study is to examine neural correlates of psilocybin effects in OCD. Neuroimaging studies of psilocybin in healthy participants have highlighted acute neural effects on areas implicated in OCD pathophysiology. A functional magnetic resonance imaging (fMRI) study of psilocybin administration in healthy controls showed acute decreases in blood oxygen level-dependent (BOLD) signal and cerebral blood flow (CBF) in fronto-temporo-parietal regions and the connectivity hubs of thalamus, putamen (striatum) and midline cortex (anterior and posterior cingulum) (38), areas which are hyperactive in OCD. Acute decreased activity in the anterior cingulate cortex (ACC) and medial prefrontal cortex (mPFC) predicted the intensity of acute subjective effects of psilocybin, and subsequent mPFC seed connectivity analysis showed that psilocybin infusion was related to acute reduced connectivity between the posterior cingulate cortex (PCC) and mPFC (39). Additionally, a preclinical study showed that psilocybin facilitates acute extinction of classically conditioned fear responses and increases hippocampal neurogenesis (40). These results may have clinical implications for psilocybin-facilitated extinction learning in OCD, given that extinction recall and associated neural activation in the vmPFC are blunted in patients with OCD (41). In summary, psilocybin appears to acutely modulate brain activity in OCD-relevant regions in healthy controls (42). In light of these findings, the present study aims to explore the neuronal correlates of potential therapeutic effects of psilocybin on OCD symptomatology, albeit in the post-dosing period. Functional imaging studies of SSRI treatment offer a precedent for treatment-induced connectivity changes linked with symptom improvement. For example, SSRI pharmacotherapy in depression (43) and OCD (44) have been shown to “normalize” aberrant connectivity in key brain regions (particularly the default mode network). Given that the medial prefrontal cortex and the striatum are centrally implicated in OCD and appear directly modulated by psilocybin, we considered frontostriatal connectivity a promising target for psilocybin’s potential theraputic effects.

### 1.2. Objectives and hypotheses

This trial aims to evaluate the feasibility of a placebo-controlled study of single-dose psilocybin in the treatment of OCD, to evaluate safety and tolerability, to provide initial evidence as to the effects of oral psilocybin on OCD symptomatology, as well as provide initial data on the neural mechanisms that may mediate psilocybin’s purported therapeutic effects on OCD.

To do so, we will use a randomized, double-blind, niacin placebo-controlled, non-crossover study design, coupled with pre-post-dosing fMRI. The choice of a single dose – as opposed to multiple administrations, as in Moreno et al. (35) – was balanced with considerations of the addition of a neuroimaging component with repeat scanning, an increase in target sample size, the use of a randomized controlled design, available budget, and the need to focus our research questions.

To investigate the effects of oral psilocybin compared to placebo on OCD symptom severity, we set the primary endpoint as 48 h post-dosing. This endpoint was selected to mitigate the possibility of sleep difficulties the night of dosing interfering with ratings and scans the next day. We hypothesize that psilocybin will lead to greater OCD symptom improvement than placebo at 48 h post-dosing. We will also track safety, tolerability, and feasibility metrics throughout the study. Additionally, we will explore the interrelationships among psilocybin-induced changes in resting-state brain connectivity and OCD symptoms. Several studies indicate that abnormal frontostriatal connectivity is central to the pathophysiology of OCD, and these regions are also affected by psilocybin. We hypothesize that at 48 h post-dosing, compared to placebo: (a) psilocybin will normalize abnormal frontostriatal functional connectivity in patients with OCD; and (b) normalization of frontostriatal functional connectivity will correlate with improvement in OCD symptomatology after psilocybin dosing. Lastly, to explore effects on constructs beyond OCD, we will examine differences between psilocybin and placebo in changes on various secondary symptom and personality measures from baseline to 48 h post-dosing.

## 2. Method

### 2.1. Trial design and setting

This study (NCT03356483) will utilize a single-site, randomized (1:1), double-blind, active placebo-controlled, non-crossover design to examine the clinical and neural effects of either a single dose of oral psilocybin (0.25 mg/kg) or active placebo-control agent (250 mg of niacin) on OCD symptoms. Consistent with prior research (35, 45), unstructured, non-directive psychological support will be provided during the dosing session, as well as during two pre-dosing preparatory visits over the two consecutive days immediately before blinded dosing, and during four integration/follow-up visits (i.e., at 48 h, 1, 2, and 12 weeks post-dosing). The choice of unstructured, non-directive psychological support (as opposed to a more structured or manualized approach) is consistent with prior psilocybin clinical research. All preparatory and integration visits last for up to 2 h each.

This study will take place from November 2018 to June 2024 at the Yale OCD Research Clinic, housed within the Clinical Neuroscience Research Unit (CNRU) of the Connecticut Mental Health Center (CMHC) in New Haven, CT. The randomized phase of the study will be conducted as a 5-day inpatient stay on this locked, research-dedicated inpatient unit. The inpatient setting is logistically viable given the number of different study procedures to be completed per study day, as well as the medical and safety oversight afforded by an inpatient setting given the novel nature of the study. The choice of an inpatient setting was also strongly suggested by the Institutional Review Board (IRB) at the time of study development for ease of safety monitoring. While the study will be conducted on an inpatient unit, we do not require study participants to present with high acute risk; in fact, such participants are excluded from participation (see Section 2.3.2). Neuroimaging will be performed at the Yale Magnetic Resonance Research Center (MRRC), located in the Anlyan Center in New Haven, CT.

This study has undergone Yale Human Research Protection Program (HRPP) review and received IRB approval. This study has received Investigational New Drug (IND) approval (IND 134406) from the U.S. Food and Drug Administration (FDA) and is conducted under Drug Enforcement Administration (DEA) Schedule 1 research regulations.

### 2.2. Population of interest and sample size

We will recruit adult participants aged 21 to 65 years old with a primary diagnosis of OCD and a Y-BOCS score of at least 19. A Y-BOCS score of 19 indicates moderate OCD symptom severity; with the criteria for a persisting diagnosis of OCD and the failure of at least one trial of standard care treatment (see Section 2.3.2), this cutoff score allows us to characterize our intended sample as treatment-refractory. Participants will be recruited irrespective of gender, sexual orientation, and ethnoracial identity. We used Optimal Design (46) to calculate sample size for a linear mixed-effects model with α = 0.05 (two-tailed), power = 0.80, and *d* = 1.5, which indicated that a total of 30 participants would be needed to detect a significant group effect at the primary endpoint per our first aim. The projected effect size of 1.5 appears reasonable given previously observed effect sizes for controlled trials of psilocybin in the treatment of major depressive disorder (45). Since this is the first study examining pre-post psilocybin resting functional connectivity changes in OCD with a placebo-controlled design, the functional connectivity analyses are largely exploratory in nature. A placebo-controlled study of psilocybin-induced connectivity changes at rest in predefined regions among 38 healthy controls detected several significant psilocybin-linked changes in mPFC connectivity (47), with Cohen’s *d*s ranging from 0.2 to 0.3. We speculate that psilocybin-linked connectivity changes may be even more dramatic in a clinical population. Assuming an attrition rate of 20%, we plan to enroll 36 participants to ensure 30 completers. Data collection is planned from November 2018 to October 2023.

### 2.3. Schedule of trial activities

**Figure 1:**
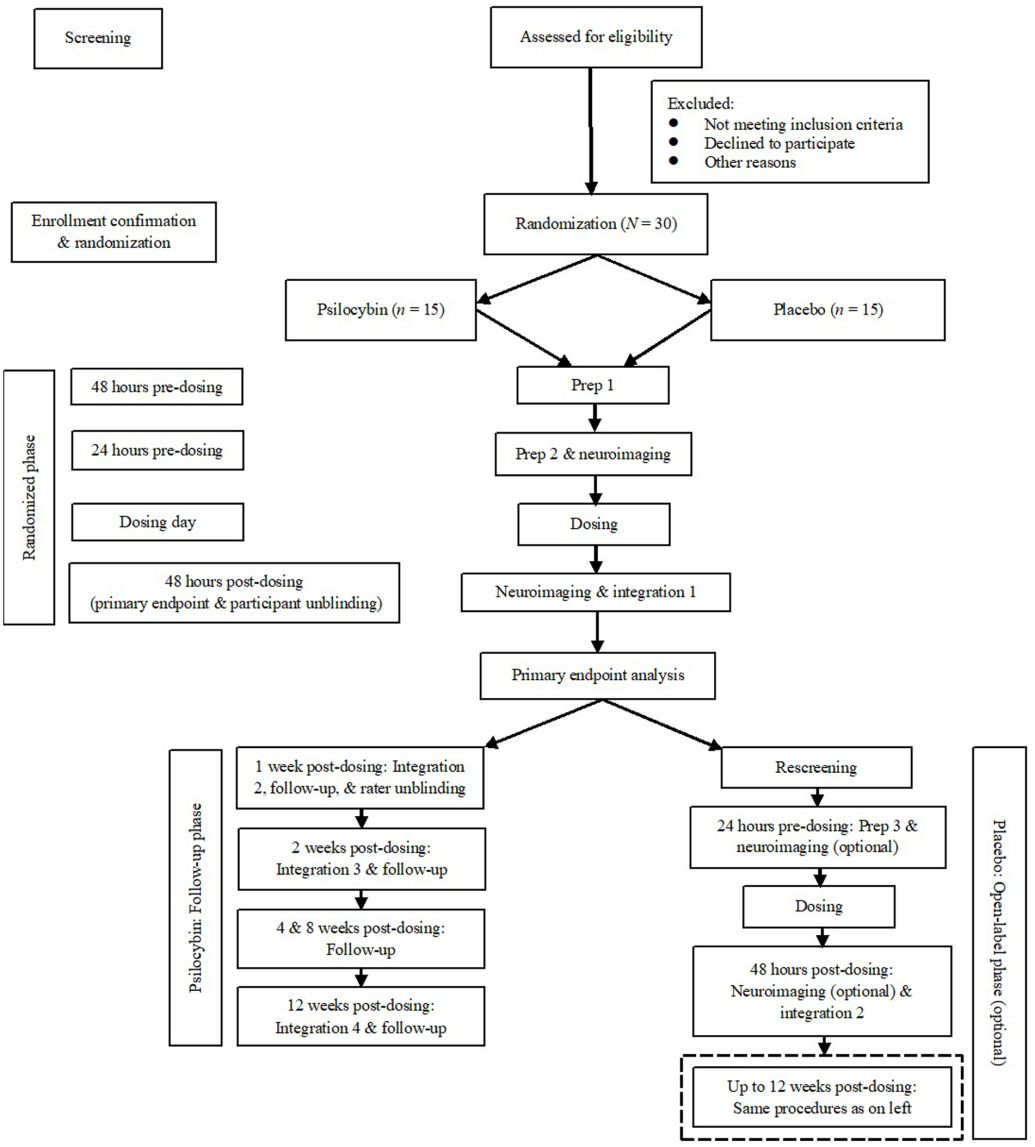
Simplified study flow diagram.

Figure 1 shows a simplified schematic of study flow from screening to termination, including the open-label phase for participants who have been randomized to placebo and unblinded.

#### 2.3.1. Recruitment

The Yale OCD Research Clinic sees a large number of potential participants with OCD annually. Recruitment strategies include community referrals (e.g., other institutional clinics/departments, local private practices, local advocacy groups or grassroots organizations) and self-referrals (e.g., through the clinic’s website, in response to online/radio/cable/TV ads or community flyers). Potential participants may also reach out through contact information provided on this study’s ClinicalTrials.gov page.

#### 2.3.2. Pre-screening, informed consent, and screening

Potential participants who express interest in this study will be directed to study personnel for phone pre-screening, informed consent, and other screening procedures. While most of these procedures/visits will be conducted in-person, some can be conducted remotely/virtually due to state/federal or site-specific COVID-19 restrictions, or because of distance.

First, potential participants will be scheduled to complete a phone pre-screen with a research assistant to review demographics, medical and psychiatric history, and screening for presence of metal implants in their body (for MRI safety). Information from this screen will be entered synchronously by study personnel directly into an electronic source document, and reviewed by the principal investigator (PI) to ascertain preliminary eligibility.

Next, participants deemed eligible at this step will then be invited for an in-person informed consent visit with the PI and/or appropriate study team members. All study procedures will be described in detail and an IRB-approved informed consent document will be reviewed and signed.

After obtaining informed consent, trained study personnel will meet with participants to complete a comprehensive psychiatric evaluation comprising a psychosocial interview, the Mini International Neuropsychiatric Interview, Version 7.0.2 (MINI-7.0.2) (48), the Y-BOCS (36, 37), the Lifetime version of the Columbia-Suicide Severity Rating Scale (C-SSRS) (47), and the obsessive–compulsive personality disorder (OCPD) and borderline personality disorder (BPD) modules from the Structured Clinical Interview for DSM-IV Personality Disorders (SCID-II) (49). In this psychiatric evaluation visit, participants were also inquired in detail about any known formal psychiatric diagnoses among immediate family members.

Participants will also complete a separate, in-person medical evaluation visit with the PI or study physician. This will comprise a medical examination, blood draws, urinary drug and pregnancy screens (if of childbearing potential), liver and thyroid function tests, and electrocardiogram (ECG). Participants will be reminded that these results will be documented on a medical chart created for them, and that they are free to discontinue participation at any time.

Inclusion criteria are:

1. Primary DSM-5 diagnosis of OCD
2. Y-BOCS score of 19 or greater
3. Failure of at least one trial of standard care treatment (medication and/or psychotherapy [CBT/ERP]) for OCD
4. English proficiency and fluency, and ability to understand the consent process and provide written informed consent
5. Willingness to sign a medical release for direct communication between research staff and external provider(s) about the participant’s treatment and medical histories
6. Non-consumption of SSRIs for at least 8 weeks at the time of randomization^1^
7. Willingness to refrain from psychiatric medications (e.g., antidepressants, first- and second-generation antipsychotics, mood stabilizers) during the study period, as well as certain other medications (e.g., anti-seizure medications, cardiovascular medications, and aldomet specifically) during the day of dosing^2^
8. Willingness to abstain from THC-containing products for study duration. A negative urinary drug screen is also required at baseline and the day of dosing.
9. A negative urinary pregnancy screen at study entry and day of dosing if of childbearing potential, and willingness to use adequate birth control for study duration
10. Having a contact person who is willing and able to be reached by the study team in the event of an emergency/crisis, and who is able to transport the participant home at the end of the inpatient stay/dosing week
11. Willingness to commit to all study procedures and visits, including inpatient stay, assessments and self-reports, neuroimaging, and being medically cleared to be discharged and transported home at the end of the dosing week

Exclusion criteria are:

1. Personal or immediate family history of schizophrenia spectrum and other psychotic disorders, bipolar I or II disorder, or major depressive disorder with psychotic features
2. Active suicidal intent
3. Unremitted Tourette syndrome
4. Autism spectrum disorder
5. OCPD or BPD
6. Current substance use disorder (except mild alcohol use disorder)
7. Unstable neurological or medical condition(s) that may render study procedures unsafe, including poorly managed diabetes, hypertension, or cardiovascular conditions, or history of seizure(s) or chronic/severe headaches
8. Any history of head injury with loss of consciousness for more than 30 min
9. Any contraindications to undergoing an MRI scan, including having metal implants or metal fragments in the body
10. Any use of psychedelic substances within the prior 12 months

#### 2.3.3. Enrollment confirmation and randomization

The PI will confirm participants’ enrollment *via* phone call and/or email after they have been reviewed successfully for all eligibility criteria. The study pharmacist alone will generate and maintain the block randomization sequence (i.e., equal number of participants in each treatment condition) using an online program to ensure blinding.

#### 2.3.4. Blinding

The following additional procedures will be undertaken to maintain blinding in this study. Participants and all research team members (including study facilitators and independent raters) will be kept blind to condition allocation before, during, and for an interval following dosing (see section 2.3.8). Psilocybin and niacin capsules are identical in terms of color, taste, and smell. Niacin (at our elected dosage of 250 mg) may also induce mild psychological (e.g., anxiety) and physiological effects (e.g., hot flushes, tingling sensations, increased heart rate) similar too low to moderate doses of psilocybin. In spite of these measures, blinding and placebo control remain a distinct challenge in psilocybin (and other psychedelic) studies due to the unique effects of psilocybin. As such, study facilitators will be attentive to and document participants’ report in regards to perceived study drug allocation anytime during dosing, up to the point of unblinding. In the event of a serious, possibly drug-related AE in which knowledge of study drug allocation is required for emergency intervention, the PI, study physician, or external emergency personnel will contact the study pharmacist. In these situations, the blind may be broken to whomever is necessary to ensure participants’ safety. The exception is the assigned independent rater, who will remain blind to allocation even under these circumstances.

#### 2.3.5. Preparation

For brevity, essential procedures for preparatory, dosing, and integration sessions in the randomized phase are concisely described from here onwards. Detailed procedures for study team members serving as study facilitators for these visits are described in a separate manual (50). While facilitator pairing will likely differ from participant to participant, the same pair of facilitators will work with each participant until study participation is completed.

During the two pre-dosing preparatory sessions, participants will meet with their assigned pair of study facilitators trained to discuss their experiences with OCD and the impact on their quality of life and functioning, life goals and aspirations, significant past experiences, and their specific hopes, expectations, fears, goals, etc. for the upcoming dosing session. In these sessions, facilitators provide psychoeducation on possible acute effects of psilocybin, and ways to ground oneself from challenging drug effects (e.g., maintaining curiosity and openness, taking the headphones and eye shades off, deep breathing, reminding participants that challenging drug effects are temporary). Further, facilitators will collaborate with participants to establish consensual boundaries around therapeutic, non-sexual touch prior to the dosing session (e.g., typically, holding the participant’s hand, or placing a hand on their arm or shoulder). Lastly, facilitators will collaborate with participants to adjust aspects of the physical setting (e.g., location of furniture, type of hanging art) to ensure physical and aesthetic comfort for the dosing day itself. At the end of the preparatory sessions, facilitators should have collaborated with participants to cultivate a presumably conducive psychological state (i.e., set) (51) for the upcoming dosing session.

#### 2.3.6. Neuroimaging

In the randomized phase, neuroimaging will be conducted at baseline 24 h pre-dosing and 48 h post-dosing. At these intervals, study personnel will accompany participants to the MRRC, where technologists will review participants’ responses on the MRI Safety Questionnaire, obtain signed informed consent for the day’s neuroimaging procedures, and conduct additional screening and safety determination if necessary (e.g., X-ray to rule out embedded metal in case of recent exposure).

MRI acquisition will be conducted using a Siemens 3 Tesla MRI scanner. Using a 64-channel head coil, the scan protocol will begin with a 3-plane localizer, scout image, and aligned 3-plane localizer that will serve as a localization series for the other acquisitions. The remaining multimodal MR pulse sequences will include: high resolution T1-weighted imaging (i.e., MPRAGE); high resolution T2-weighted imaging; spin echo field maps acquired in opposing phase encoding directions prior to each fMRI session; multi-band BOLD gradient echo planar imaging at rest; diffusion weighted imaging (DWI), and arterial spin labeling (ASL). The T1 and T2 acquisitions are set to use an “Adjust Volume” that matches the size and positioning of the spin echo field maps and resting state fMRI scans. Total MR acquisition may last between 1.5 and 2 h depending on whether some series must be re-acquired due to confounds/artifacts.

#### 2.3.7. Dosing

Early in the morning of the dosing day (approximately 7 am), participants will be allowed to consume a light breakfast free of milk/dairy or caffeine, so as to not affect drug absorption or effects. By approximately 10 am, the participant will be settled into a study treatment room on the CNRU, accompanied by the study facilitators, to begin the dosing session. One facilitator will be designated beforehand to monitor the participant’s vitals (blood pressure, heart rate, temperature) and subjective units of distress (SUDS; from 0 to 100) at baseline prior to ingestion of the drug capsule, as well as at subsequent intervals until the end of the dosing session (i.e., 15 and 30 min post-dosing, then every 30 min after until 2 h post-dosing, then every hour after until end of dosing session). Vital sign monitoring will continue beyond the dosing session at regularly scheduled CNRU shift checks for the remainder of the inpatient stay.

The study pharmacist will prepare and deliver the study drug by 10 am, and remain in the room until the participant has ingested the study drug. During the first few hours, participants will be encouraged to lie comfortably in bed with the headphones and eye shades on, and listen to a standardized music playlist designed to mimic the prototypical arc of a psilocybin experience. The music helps eliminate extraneous distractions and facilitate “dropping into” the dosing experience. While there are likely idiosyncrasies to each psilocybin experience, most follow a trajectory of ‘ascent’ (0 to 1.5 h post-dosing; onset of drug effects), ‘peak’ (1.5 to 3.5 h post-dosing; greatest intensity of drug effects, sometimes in one or more ‘waves’), and ‘descent’ (3.5 to 5 h and beyond; subsiding of acute drug effects).

Regardless of condition assignment, study facilitators will attend to participants’ physical and psychological needs and safety for the entire dosing session (50). Specifically, facilitators will attend to participants’ reactions to any arising internal experiences, as well as support them in engaging openly with these experiences in a non-directive, non-judgmental, compassionate, and empathetic manner. At least one facilitator must be physically present in the room with the participant at all times. If appropriate, facilitators will employ grounding strategies and/or consensually agreed therapeutic touch to alleviate participants’ distress. In the event that participants do not respond to these strategies and/or if participants are endangering themselves or others, the PI/study physician will prescribe and administer a one-time anxiolytic “rescue” medication (benzodiazepine) or intervene otherwise as appropriate.

The dosing session is expected to last up to 6 or more hours. Participants will be required to stay in the dosing room for at least 4 h, or until the effects of the ingested drug have worn off, whichever is longer. At the end of the session, participants will be asked about any residual drug effects or other AEs. Each participant will be expected to remain under observation in the dosing room until the participant, study facilitators, and PI/study physician all agree that the participant’s perception, cognition, functioning, and judgment have returned to their baseline levels or are no longer significantly impaired by the drug. Participants will be encouraged to document in any mode of creative expression their recollection and initial reflections of their dosing experience prior to the first integration session 48 h post-dosing. This may offer participants the opportunity to independently consolidate any emerging insights, and provide content to explore during integration sessions. Participants were also discouraged from discussing their dosing experience at length with family or friends over the phone, or in-person with staff or other patients on the unit, and to reserve such discussion for the integration sessions. Thereafter, participants will be allowed to have dinner and retire to their room on the unit.

If participants experience or report any persisting drug effects or SAEs, including acute suicidality, they will continue to be monitored on the inpatient unit, with follow-up interventions and procedures (e.g., transfer to emergency department/hospital, longer-term psychiatric hold on the unit, or withdrawal and referrals for treatment) implemented as necessary.

#### 2.3.8. Unblinding

Participant and facilitator blinding are maintained only until the primary endpoint of 48 h post-dosing in the randomized phase, to facilitate scheduling of subsequent open-label dosing for participants assigned to placebo (see Section 2.3.10.1). Specifically, unblinding will occur at the start of the 48 h post-dosing integration visit, which will always occur after the 48 h post-dosing ratings and fMRI scan. Independent raters will remain blinded until after the one-week post-dosing ratings are completed, which will be past the primary endpoint, thus not impacting primary outcomes.

#### 2.3.9. Integration/follow-up

Participants will complete their first integration session with their assigned study facilitators 48 h post-dosing, prior to being discharged. Participants randomized to placebo will be offered the opportunity to return for open-label psilocybin dosing (0.25 mg/kg) approximately 2 weeks later (see section 2.3.10.1). Participants randomized to psilocybin will go on to complete three more integration visits 1, 2, and 12 weeks post-dosing, for a total of four integration visits.

Over the course of these integration sessions, facilitators will attend to, encourage, and support participants in describing and processing their psilocybin experience in a detailed manner (50). These discussions are meant to be open-ended, non-directive, unstructured, and participant-led, with the goal of aiding participants in consolidating any emergent insights from the dosing session and beyond, so as to utilize these insights as potential guiding principles for managing and living with OCD moving forward. During these integration sessions, facilitators will encourage participants to reference any journal entries, drawings, or other modes of creative expression that they may have engaged in post-dosing, to aid in discussing their psilocybin experience. During these sessions, facilitators will also document any new or persisting drug effects or AEs, and provide appropriate interventions if needed.

#### 2.3.10. Optional trial components

##### 2.3.10.1. Open-label phase

Participants randomly assigned to the placebo condition will be eligible for continued study participation in the open-label phase, which will be scheduled approximately 2 weeks after unblinding. These participants will be re-screened after unblinding to ensure that they still meet eligibility criteria, and verbally probed to ensure that they still consent to participate in the open-label phase. The open-label phase of the study will follow nearly identical procedures to those for participants randomly assigned to the psilocybin condition in the randomized phase (i.e., preparatory, dosing, and integration/follow-up visits). The only exceptions are that neuroimaging will be optional during the open-label phase, and there will only be one instead of two preparatory sessions (hence fewer nights of stay on the inpatient unit), given the already substantial interaction with study facilitators. Participants will be re-assessed on all study measures based on the same schedule as in the randomized phase. Participants need not complete any portion of the open-label phase, including the several subsequent follow-up visits, for their participation in the study to be considered completed. Data will be collected from all participants during the open-label phase for descriptive purposes but will be included only in secondary analyses of psilocybin’s effects on OCD and related symptomatology.

##### 2.3.10.2. Qualitative interview

An interview will be conducted by an independent interviewer one-month post-psilocybin dosing with all participants. This is optional, and participants will need to provide additional consent. This interview seeks to gather qualitative data about participants’ psilocybin experiences, insights, and any changes in OCD symptoms, quality of life, and functioning 1 month post-dosing.

### 2.4. Outcomes

#### 2.4.1. Clinical outcomes

##### 2.4.1.1. Primary outcomes

The primary outcome measures are the acute version of the clinician-administered Y-BOCS (AY-BOCS) (52) and the self-report Visual Analog Scale (VAS) for OCD symptoms (53), both of which assess OCD symptom severity over the past 24 h. These will be administered by independent raters at baseline and 24 and 48 h post-dosing. The Y-BOCS will also be administered by the same raters at baseline and 1, 2, 4, 8, and 12 weeks post-dosing.

Safety outcomes are included as primary outcomes as well. Safety measures assessed only during dosing include vitals and SUDs, and those assessed throughout the study duration include the Since Last Visit version of the C-SSRS (54), AEs, and any required changes to psychiatric or non-psychiatric medication regimens. The Since Last Visit version of the C-SSRS (54) assesses suicidal ideation severity, ideation intensity, and suicidal behavior severity (including non-suicidal, self-injurious behaviors) since the last assessment interval (i.e., the last study visit). AEs are defined as any untoward physical, social, economic, or psychological occurrence affecting participants, including any abnormal laboratory finding, symptom, reaction, or disease. An AE does not necessarily have a causal relationship with study procedures, and are graded as mild, moderate, or severe in this study. Severity of an AE is considered distinct from its “seriousness.” An AE is considered serious (i.e., SAE) if it: (1) is life-threatening; (2) results in hospitalization, disability/incapacity, a congenital anomaly or birth defect, or death; (3) requires medical or surgical intervention to prevent aforementioned outcomes; or (4) adversely affects the risk/benefit ratio of the study. Therefore, a severe AE is not necessarily an SAE.

To evaluate feasibility and tolerability, the following metrics will be tracked and reported. Specifically, we will report the total number of participants screened, found to be eligible, enrolled, had their enrollment confirmed, and randomized. We will also report on the total number of participants who were found to be ineligible, who voluntarily withdrew from participation prior to randomization, and who were excluded from participation due to safety concerns. In order to evaluate the restrictiveness of our eligibility criteria, we will report the number of participants excluded on the basis of different sets of criteria. Additionally, we will report the follow-up rates (i.e., number of participants who completed follow-up divided by the number of enrolled participants) by treatment arm for all study visits up to 12 weeks post-dosing. Number and percentage withdrawal from the trial with reasons for withdrawal will be summarized at each follow-up and by treatment arm. We will also summarize the number and percentage of participants with missing data for primary and secondary outcomes as a whole and by treatment group and follow-up intervals up to 12 weeks post-dosing. We will employ descriptive statistics and, if possible, logistic regressions to compare baseline characteristics of completers vs. participants with missing data at each follow-up interval.

##### 2.4.1.2. Secondary outcome

The secondary outcome in this study is resting-state brain connectivity, which will be assessed at baseline and 48 h post-dosing.

##### 2.4.1.3. Exploratory outcomes

Exploratory outcomes are assessed by clinician-rated or self-report measures that will be administered at baseline, immediately after dosing, and/or at varying follow-up intervals up to 12 weeks post-dosing. These measures include:

1. Obsessive–Compulsive Inventory-Revised (OCI-R) (55)
2. Obsessive–Compulsive Trait Core Dimensions Questionnaire (OC-TCDQ) (56)
3. Padua Inventory (PI) (57)
4. State–Trait Anxiety Inventory (STAI) (58)
5. Montgomery-Åsberg Depression Scale (MADRS) (59)
6. Beck Depression Inventory, Second Edition (BDI-II) (60)
7. Alcohol Use Disorders Identification Test (AUDIT) (61, 62)
8. Drug Use Disorders Identification Test (DUDIT) (63)
9. Self-Reported Nicotine Use (SRNU) (64)
10. Obsessive Beliefs Questionnaire-44 (OBQ-44) (65)
11. Thought-Action Fusion Scale (TAFS) (66)
12. Responsibility Attitude Scale (RAS) (67)
13. Big Five Aspects Scale (BFAS) (68)
14. Quality of Life Enjoyment & Satisfaction Questionnaire-Short Form (Q-LESQ-SF) (69)
15. Clinical Global Impression (CGI) – Clinician-Administered and Self-Report (70, 71)
16. Sheehan Disability Scale (SDS) (72)
17. Positive and Negative Affect Schedule-Expanded Form (PANAS-X) (73)
18. Mystical Experience Questionnaire (MEQ) (74)
19. Challenging Experience Questionnaire (CEQ) (75)
20. 5-Dimensional Altered States of Consciousness Rating Scale (5D-ASC) (76)
21. Persisting Effects Questionnaire (PEQ) (77)
22. Nature Relatedness Scale (NRS) (78)
23. Pro-Environmental Behavior Scale (PEBS) (79)
24. Individual Differences in Anthropomorphism Questionnaire (IDAQ) (80)
25. Mind–Body Dualism Scale (MBDS) (81)
26. Inclusion of Other in the Self Scale (IOS) (82)
27. Ethics Position Questionnaire (EPQ) (83)
28. Revised Life Orientation Test (LOT-R) (84)
29. Schedule for Meaning in Life Evaluation (SMiLE) (85)
30. Behavior Identification Form (BIF) (86)
31. Pittsburgh Sleep Quality Index (PSQI) (87)
32. Reactions to Research Participation Questionnaire (RRPQ) (88, 89)
33. University of Rhode Island Change Assessment (URICA) (90)
34. Utilization of Facility and Emergent Care (UFEC) (91)

Facilitators will administer paper self-reports of acute drug effects immediately post-dosing. The rest of the self-reports will be administered by research assistants *via* a link to the electronic data capture system on a tablet.

#### 2.4.2. Evaluation of success of blinding

Immediately prior to unblinding of participants at 48 h post-dosing, participants will be verbally probed to name the study arm they believe they were assigned to, with this response documented on the visit note.

### 2.5. Facilitator competencies

A separate manual (50) describes expected core competencies, skills, and theoretical orientation of study facilitators, as well as guiding principles for psilocybin dosing session facilitation. Because there is no structured program of psychological support tested in this trial, treatment fidelity is not assessed. Instead, study facilitators are expected to verify that they have thoroughly reviewed and understood the contents of the facilitation manual, and to continuously process and discuss their interactions with participants in study team meetings, to ensure that they are facilitating sessions according to guidelines in the manual.

### 2.6. End of trial

End of trial is defined as the time point at which the database is locked from data entry, which may be the same as or longer than the last time point of data collection (i.e., at the last follow-up visit with the final completer).

### 2.7. Trial monitoring

The PI will form a Data and Safety Monitoring Board (DSMB) comprising: (1) a psychiatrist with extensive OCD experience; (2) a cardiologist or internal medicine specialist; and (3) a general physician. The PI and DSMB will be responsible for safety oversight by monitoring the data, assuring protocol compliance, and conducting safety reviews every 6 months, including when reapproval of the protocol is sought. During the review process, the PI in collaboration with the DSMB will evaluate whether the study should continue unchanged, require modification or amendment, or close to enrollment; the latter two decisions will be communicated as soon as possible to the IRB/Human Investigations Committee (HIC). Either the PI, DSMB, or the IRB/HIC will have the authority to stop or suspend the study, or require modifications.

Additionally, the PI will periodically review the collection, storage, and distribution practices associated with the clinical data bank, and determine whether changes to enhance confidentiality and privacy are required. In terms of clinical monitoring, the research team will follow the Yale HIC’s guidelines for attribution and grading the severity of AEs, including determining whether an AE meets criteria for an SAE, and necessary follow-up IND reporting.

If the trial is prematurely stopped and terminated, the PI will be required to promptly inform active study participants, and provide appropriate therapy referrals if necessary and follow-up. All procedures and requirements pertaining to retention and storage of documents will still be observed. All other study materials will be treated in accordance with federal and state regulations.

### 2.8. Data collection, storage, and security

This study will be conducted within organizations fully bound by and compliant with HIPAA policies and strict research requirements. Data will be recorded in both written and/or electronic formats. Paper source documents/charts will be stored in locked filing cabinets in a locked office, while electronic records and data will be stored as password-protected files in secured computer(s) or server(s), all with access restricted to the PI and relevant study team members with appropriate training. Personal identifiers and protected health information (PHI) will be restricted to name, address, email address, phone number, birth date, and dates of study visits. PHI can be accessed only by the PI and specified research staff approved by the PI. No personally identifying data will appear in the digital data files or dissemination efforts (e.g., presentations, publications) resulting from this study. A Certificate of Confidentiality (CoC) has been obtained from the FDA for this study, providing additional protection for confidential participant data.

### 2.9. Statistical analysis

We will conduct linear mixed modeling (LMM) with restricted maximum likelihood estimation (to handle missing data) with appropriate alpha correction in SPSS to test our first hypothesis that psilocybin, compared to placebo, will improve OCD symptoms at the primary endpoint of 48 h post-dosing on the VAS for OCD symptoms and AY-BOCS. Treatment, time, and all interactions will be modeled as fixed effects. Participants will be modeled as a random effect. The time factor will be specified as baseline to 48 h post-dosing. Alpha-corrected *t*-tests will be used to probe interaction effects.

MRI preprocessing and group difference statistics will be evaluated using standard regression and general linear modeling approaches in a general-purpose MRI analysis package (QuNex), a processing platform that incorporates several state-of-the art neuroimaging tools (FSL, FreeSurfer, AFNI, SPM, PALM, and HCP-MPP) and thus offers comprehensive multimodal MRI analytic capabilities (92). To test the secondary hypothesis that at 48 h post-dosing, psilocybin, compared to placebo, will normalize frontostriatal connectivity at rest, we will conduct similar LMMs to examine the effect of psilocybin on activation of the corresponding regions. To test the secondary hypothesis that normalization of activity in these regions at 48 h post-dosing will correlate with clinical improvement in OCD symptoms (i.e., on the VAS for OCD symptoms, AY-BOCS, and Y-BOCS), we will conduct a correlational analysis with these variables.

Exploratory analyses of change in other secondary outcomes from baseline to 48 h post-dosing will be conducted using similar LMMs. Safety data will be presented as descriptive statistics. Data from the one-month post-dosing interviews will be qualitatively analyzed *via* interpretative phenomenological analysis (IPA) (93).

### 2.10. Ethics and dissemination

The trial was approved by the Yale University HIC (#2000020355) and was registered with ClinicalTrials.gov (NCT03356483). Protocol modifications will be reviewed and approved by the Yale University HIC and updated on ClinicalTrials.gov. Reconsent will be sought from participants, if necessary. We will submit a manuscript describing the primary outcome for publication in a peer-reviewed journal. Manuscripts for secondary outcomes and qualitative data will also be submitted as appropriate. Lastly, the results from this trial may be publicized *via* other media.

## 3. Discussion

This study is the first randomized, placebo-controlled trial of psilocybin for OCD in the world. If psilocybin administration is well-tolerated and leads to a rapid and substantial reduction in OCD symptoms, it would represent an impactful advance in our ability to treat severe and refractory OCD. At the same time, the neurobiological mechanisms underlying the robust effects of psilocybin remain poorly understood. Thus, the present study may also pave the way for future studies of neurobiological mechanisms of OCD that may respond to psilocybin, in order to further optimize existing and innovative pharmacotherapies.

Strengths of this study include the use of a randomized, placebo-controlled, double-blinded design, and the ability to directly compare primary and neuroimaging outcomes between groups in order to inform future efficacy and clinical neuroimaging trials. On the other hand, there are a few limitations to this study. As mentioned, it may be hard to maintain blinding due to the unique effects of psilocybin. As such, future designs can consider non-pharmacological alternatives for the active control group, such as virtual reality-based simulations of psychedelic experiences (94). Additionally, it is not known whether other preparations of psilocybin (e.g., dried psilocybin-containing mushrooms, with additional alkaloids such as baeocystin) than the version used in this trial (i.e., synthetic psilocybin) would show the same proposed effects. This is also a single-site study in an inpatient setting, with a small intended sample of participants who are treatment-refractory and at low risk for suicide. These characteristics likely place limits on generalizability of eventual findings (e.g., to outpatient studies, to the larger population of individuals with OCD). Further, 48 h post-dosing may not be the ideal endpoint to observe neural effects of psilocybin in OCD; future research is needed to identify optimal follow-up intervals. Another limitation is the choice of weight-based psilocybin dosing, which may not be sufficient for certain lower-weight individuals to achieve hypothesized clinical and neural effects. Lastly, while a non-directive, unstructured approach to psychological support will be implemented in this study, we did not include any measures assessing how psychological support is actually administered (e.g., the frequency and type of directive interventions that were applied to relieve psychological distress during dosing). This and other extrapharmacological factors (e.g., treatment expectancy, perceptions of setting) should be assessed and analyzed as potential moderators of treatment response in future studies.

## Author contributions

BK and CP conceptualized this research and finalized the research design, study procedures, and statistical analytical approach. TC drafted the initial version of the manuscript. All authors provided revisions and contributed to the final version of the manuscript.

## Funding

This work was supported by a grant from the Yale Center for Clinical Investigation (YCCI), grants from the Heffter Foundation and the Turnbull Family Foundation, and by the State of Connecticut through its support of the Ribicoff Research Facilities at the Connecticut Mental Health Center. The Usona Institute provided the investigational drug. The funding organizations were not involved in the design and conduct of this trial, and will not be involved in the analysis, interpretation, or dissemination of the data.

## Conflict of interest

The authors declare the following conflict of interests. TC serves as a continuing faculty member in the Psychedelic-Assisted Therapy Training Program offered by Integrative Psychiatry Institute (IPI), and consults for Transcend Therapeutics. CP serves/has served as a consultant for Biohaven, Teva, Lundbeck, Brainsway, Ceruvia Lifesciences, Transcend Therapeutics, Nobilis Therapeutics, and Freedom Biotech, receives royalties and/or honoraria from Oxford University Press and Elsevier, and has filed a patent on the use of neurofeedback in the treatment of anxiety, which is not relevant to the current work. BK is co-founder and Chief Scientific Advisor for Transcend Therapeutics and has consulted for Ceruvia Lifesciences and Lobe Sciences. CP and BK have filed a patent on the use of psilocybin in the treatment of obsessive–compulsive disorder (#US17/466,111).

The remaining authors declare that the research was conducted in the absence of any commercial or financial relationships that could be construed as a potential conflict of interest.

## Publisher’s note

All claims expressed in this article are solely those of the authors and do not necessarily represent those of their affiliated organizations, or those of the publisher, the editors and the reviewers. Any product that may be evaluated in this article, or claim that may be made by its manufacturer, is not guaranteed or endorsed by the publisher.

## Footnotes

^1^Any necessary medication tapering will be done between participants and their prescriber(s), without the study team’s involvement.

^2^Exceptions are benzodiazepines as needed up to 3 days before dosing, and prescribed opiate pain medication or over-the-counter non-narcotic pain medication as needed at any time. The PI may also prescribe zolpidem for insomnia as needed during the inpatient stay. Should participants’ external providers recommend starting a new course of exclusionary medication(s), participants must notify the study team.
